# Differentiation of Cells Isolated from Human Femoral Heads into Functional Osteoclasts

**DOI:** 10.3390/jdb10010006

**Published:** 2022-01-18

**Authors:** Daniel R. Halloran, Brian Heubel, Connor MacMurray, Denise Root, Mark Eskander, Sean P. McTague, Heather Pelkey, Anja Nohe

**Affiliations:** 1 Department of Biological Sciences, University of Delaware, Newark, DE 19716, USA; dhallor@udel.edu (D.R.H.); brianph@udel.edu (B.H.); cmacm@udel.edu (C.M.); smctague@christianacare.org (S.P.M.); 2 Orthopedic Surgery, ChristianaCare Hospital, Wilmington, DE 19801, USA; droot@christianacare.org; 3 Delaware Orthopaedic Specialists, Newark, DE 19713, USA; markeskander77@me.com; 4 Orthopedic Surgery, ChristianaCare Hospital, Newark, DE 19716, USA; Heather.L.Pelkey@christianacare.org

**Keywords:** bone, osteoclasts, TRAP, Cathepsin K, RAW 264.7 cells, osteoarthritis, osteoporosis, M-CSF, RANKL

## Abstract

Proper formation of the skeleton during development is crucial for the mobility of humans and the maintenance of essential organs. The production of bone is regulated by osteoblasts and osteoclasts. An imbalance of these cells can lead to a decrease in bone mineral density, which leads to fractures. While many studies are emerging to understand the role of osteoblasts, less studies are present about the role of osteoclasts. This present study utilized bone marrow cells isolated directly from the bone marrow of femoral heads obtained from osteoarthritic (OA) patients after undergoing hip replacement surgery. Here, we used tartrate resistant acid phosphatase (TRAP) staining, Cathepsin K, and nuclei to identity osteoclasts and their functionality after stimulation with macrophage-colony stimulation factor (M-CSF) and receptor activator of nuclear factor kappa-β ligand (RANKL). Our data demonstrated that isolated cells can be differentiated into functional osteoclasts, as indicated by the 92% and 83% of cells that stained positive for TRAP and Cathepsin K, respectively. Furthermore, isolated cells remain viable and terminally differentiate into osteoclasts when stimulated with RANKL. These data demonstrate that cells isolated from human femoral heads can be differentiated into osteoclasts to study bone disorders during development and adulthood.

## 1. Introduction

The Formation of the skeleton during embryogenesis and early stages of development is crucial for proper protection, support, and function of human organ systems [1,2,3,4]. This process is governed by two major bone cell types, which include osteoblasts and osteoclasts [5,6,7,8,9,10]. Osteoblasts are mononucleated cells responsible for forming new bone and derive from mesenchymal stem cell (MSC) progenitors [11,12,13,14]. Contrarily, osteoclasts are multinucleated immune cells that derive from hematopoietic stem cells (HSCs) and resorb old or damaged bone [8,10,15,16,17,18]. The crosstalk between these two cell types is critical for the formation of early structures, such as digits, and bone homeostasis throughout adulthood [1,19,20]. Indeed, about 10% of the adult human skeleton is renewed each year and is dependent on the balance between osteoblasts and osteoclasts [5,8,9,21]. However, as humans age, the balance shifts toward bone resorption, and may lead to bone disorders such as osteopenia and osteoporosis (OP) [21,22,23,24,25,26]. Thus, elucidating the precise mechanisms that are responsible for this shift in homeostasis is of the utmost importance.

**Figure 1:**
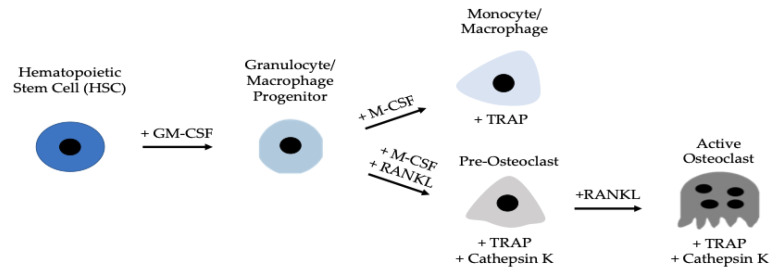
Development of an active osteoclast. Hematopoietic stem cells (HSCs) differentiate into GMPs, which express c-fms receptors that M-CSF ligands can bind. After binding, GMPs differentiate into monocyte/macrophage precursors, that further differentiate into pre-osteoclasts and active osteoclasts when RANKL binds to RANK receptors. Active osteoclasts are multinucleated and express TRAP and Cathepsin K.

Osteoclastogenesis and the activity of osteoclasts are reliant on osteoblasts [16,27,28,29,30]. Osteoblasts secrete two proteins that are necessary for osteoclast function and differentiation: macrophage colony stimulating factor (M-CSF) and receptor activator of nuclear factor kappa-β ligand (RANKL) [31,32,33]. As osteoblasts secrete M-CSF, this ligand subsequently binds to colony-stimulating factor-1 (c-fms) receptors located on granulocyte/macrophage progenitors (GMPs) or peripheral blood mononuclear cells (PBMCs) [34,35,36]. Here, GMPs differentiate into monocyte/macrophage cells, which are considered osteoclast precursors [37]. Simultaneously, osteoblasts are secreting RANKL, which will bind to RANK receptors expressed on the cell surfaces of monocytes/macrophages [38,39]. The monocytes/macrophages can then terminally differentiate into functional osteoclasts, which will be responsible for resorbing bone (Figure 1). The M-CSF and RANKL signaling pathways promote cell differentiation, survival, and proliferation by activating the Akt, Erk, NF-κB, and MAPK pathways [18]. It has previously been reported that murine RAW264.7 cells of the monocyte/macrophage lineage respond to these factors and can be used as a model for studying osteoclastogenesis [16,27]. However, the effects of M-CSF and RANKL in bone marrow cells isolated directly from human femoral heads remains unclear.

Osteoclasts are expressed predominantly during bone resorption, making them difficult to isolate in high concentrations so as to study osteoclastogenesis [40,41,42]. The isolation process becomes more difficult when obtaining osteoclasts or pre-osteoclasts directly from the bone marrow of patients with bone disorders, such as OP or osteoarthritis (OA). Moreover, previous studies suggest that while osteoclasts may be isolated, they are obtained at low concentrations, especially when terminally differentiated [42,43,44,45]. Furthermore, as these cells do not proliferate, it is difficult to maintain and keep them viable [43,44,45]. These cells also undergo apoptosis rapidly when isolated, posing many challenges when maintaining them [46]. To combat these challenges, previous studies have utilized PBMCs obtained from human blood [32,36,37,41,47]. As these cells are consisted of monocytes/macrophages, this method is a powerful technique to study osteoclastogenesis. However, less data are available regarding cells isolated directly from the bone marrow of humans, which may provide additional insight into the function of osteoclasts in the bone microenvironment. Therefore, the development of a reliable and usable model that isolates osteoclast precursors that become functional osteoclasts directly from the bone microenvironment is crucial. A current option is human femoral heads obtained after hip replacement surgery, as they are readily available and are representative when studying human bone disorders.

While RAW264.7 cells are efficient and reliable to study osteoclastogenesis, they are a murine cell line and may not be representative of human disorders. Therefore, the utilization of cells isolated from human femoral heads may be a better model to study osteoclast activity. Here, we demonstrate that cells isolated directly from the bone marrow of OA patients after undergoing hip replacement surgery can be differentiated into osteoclasts that are viable and functional. We showed that pre-osteoclasts do not differentiate readily when stimulated with only M-CSF, but differentiate frequently when exposed to both M-CSF and RANKL. Furthermore, we demonstrated that isolated cells stained positive for tartrate-resistant acid phosphatase (TRAP) and Cathepsin K, which are enzymes expressed by osteoclast precursors and osteoclasts during bone resorption. While 60% of the cells stimulated with RANKL differentiated into multinucleated osteoclasts, 92% and 83% of the cells stained for Cathepsin K and TRAP, respectively. These data demonstrate that our cell population was predominantly osteoclasts and pre-osteoclasts. These data indicate that cells isolated from the femurs of diseased patients can be grown and differentiated to better understand the imbalance between osteoblast and osteoclast activity. In summary, these results and methods will help future research uncover potential therapeutics that are desperately needed to treat bone disorders.

## 2. Materials and Methods

### 2.1. Femoral Head Retrieval

Human femoral heads were obtained from ChristianaCare hospitals (Wilmington, DE, USA and Newark, DE, USA). The femoral heads were collected from 10 male OA and 6 female patients that underwent total hip replacement surgery. The age range of the patients at the time of surgery was 49–83 years. Following surgical removal, the samples were stored in a 4 °C refrigerator and collected for this study on the same day.

### 2.2. Cell Isolation from Femoral Heads

**Figure 2:**
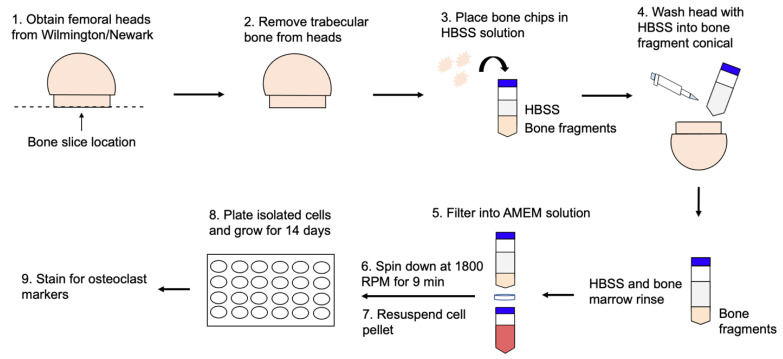
Femoral heads from patients diagnosed with OA or OP were obtained from ChristianaCare in Newark, DE or Wilmington, DE. The trabecular bone was removed from the heads and placed in α-MEM. The interior surface of the bone was washed with HBSS. After, the α-MEM and HBSS solutions were filtered with a 70 µm filter. The solution was spun down at 1800 RPM for 9 min at 4 °C to form a cell pellet. The pellet was resuspended in 5 mL of α-MEM and cells were plated at a density of 1 × 10^5^ cells/mL supplemented with α-MEM and 25 ng/mL M-CSF. Cells were grown and subjected to experimentation.

Trabecular and cortical bone were extracted from the femoral heads and placed into a 50 mL conical tube containing 10 mL of Hanks′ Balanced Salt Solution (HBSS). Followed by bone removal, the bone marrow of the samples was washed with additional HBSS to collect bone marrow cells. After the cells settled for 2−3 min, the solution was filtered through a 70 µm cell filter into a separate 50 mL falcon tube containing 5 mL of alpha modified Minimum Essential Medium Eagle (α-MEM; Caisson Labs, Smithfield, UT, USA, Cat# MEL08-500ML), supplemented with 10% fetal bovine serum (FBS; Gemini Bioproducts, West Sacramento, CA ,USA), 1% penicillin/streptomycin (pen/strep; Fisher Scientific, Pittsburg, PA, USA), and 1% antibiotic/antimycotic (anti/anti; Gemini Bioproducts, West Sacramento, CA, USA). The filtered solution was centrifuged at 1800 revolutions per minute (RPM) for 9 min at 4 °C. The cell pellet was resuspended in α-MEM and plated into 12-well or 24-well plates at a cell density of 1 × 10^5^ cells/mL (Figure 2).

### 2.3. Cell Culture

RAW264.7 cells, which are of the monocyte/macrophage lineage, were obtained from American Type Culture Collection (Manassas, VA, USA). To differentiate these cells into osteoclasts, cells were plated in 12-well plates with 18 mm diameter rounded coverslips (Catalog #CS-R18-100, Amscope, Irvine, CA, USA) at a density of 1 × 10^3^ cells/cm^2^. RAW264.7 cells were grown for 24 h in Dulbecco’s Modified Eagle Medium (Cat# 23-90-013-PB, DMEM, Krackeler Scientific, Albany, NY, USA) containing 0.1 mg/L streptomycin, 100 U/L penicillin, 2 mM L-glutamine (Gemini Bioproducts, West Sacramento, CA, USA), 1.8 g/L sodium bicarbonate, and 1 mM sodium pyruvate (Cellgro, Herndon, VA, USA). The RAW 264.7 cells were then treated with 10 ng/mL of RANKL (Sino Biological, Beijing, China) for 5 days. The cells were then stained using a Tartrate Resistant Acid Phosphatase (TRAP) kit and were imaged to identify osteoclasts.

Extracted cells from femoral heads were plated and supplemented with α-MEM, along with 10% FBS, 1% penicillin/streptomycin, 1% antibiotic/antimycotic, and 25 ng/mL M-CSF. The cells were incubated at 37 °C with 5% CO_2_ for five days. On day five, the media were replaced, and cells were stimulated with 50 ng/mL RANKL and 25 ng/mL M-CSF (Sino Biological, Beijing, China) or left unstimulated (M-CSF only) for five days. Five days later, the media were replaced, and cells were restimulated with RANKL or left unstimulated. On day 14, the stimulation was terminated, and the media were removed from the wells.

### 2.4. Optimization of Differentiating Pre-Osteoclasts into Osteoclasts

To determine the optimal conditions to differentiate pre-osteoclasts into functional osteoclasts, various cell densities and concentrations of RANKL/M-CSF were supplemented in the cell culture. After the primary cells were isolated from the human femoral heads, they were subjected to four experimental conditions. The cells were then stained for TRAP and were imaged to identify osteoclasts.

### 2.5. Tartrate Resistant Acid Phosphatase (TRAP) Staining

To observe if the cells isolated from the human femoral heads could be differentiated into osteoclasts, they were stained for TRAP, an enzyme highly expressed by osteoclasts [48]. After removing the media from the wells, the cells were washed three times with 1x phosphate buffered saline (PBS). The cells were then fixed with 4.4% paraformaldehyde (PFA, pH 7.2; Sigma-Aldrich, St. Louis, MO, USA) for 15 min at room temperature. After, the cells were washed three times with DiH_2_O and stained for TRAP using an acid leukocyte phosphatase kit (Cat# 387-1KT, Signa-Aldrich, St. Louis, MO, USA) following the manufacturer’s protocol. The cells were washed three times with DiH_2_O, and the nuclei were counterstained with Hematoxylin Gill No. 3 (Cat# 387-1KT, Signa-Aldrich, St. Louis, MO, USA) for 2−3 min. The wells were washed several times with alkaline water and left to dry in the dark for at least two days. Osteoclasts were identified as having at least three nuclei and visible TRAP stains. As macrophages can express TRAP, only cells with more than three nuclei and positive TRAP staining were included in the osteoclast count.

At least 10–15 random images of each experimental condition were obtained using the Zeiss Axiovert 10 microscope (Nohe Laboratory, University of Delaware, Newark, DE, USA) with the 20×/12 Achrostigmat objective, providing at least 20 images for each OA patient (*N* = 10; 5 male and 5 female samples). The experiments conducted for each patient were performed in triplicate. Representative images of the total cell count and osteoclast count are displayed underneath each respective bar graph. The images were processed and counted with ImageJ (NIH, Bethesda, MD, USA).

### 2.6. Cell Viability and Proliferation Assay

Primary cells isolated from human femoral heads were treated with a Green Live/Dead (Catalog #6342, Immunochemistry Technologies, Bloomington, MN, USA) stain for viability and Calcein-AM-red-orange (Catalog #C34851, Thermo Fisher Scientific) stain for proliferation once a day for 5 days. The cells were counted each day and were considered viable if they stained positive for Calcein-AM but did not stain positive for green, as the green can only penetrate the cell membranes of dead cells. Images and counts were collected using the Nikon Eclipse TE300 epifluorescence microscope (15 Innovation Way, University of Delaware, Newark, DE, USA). Experiments were conducted with three patients and were repeated in triplicate.

### 2.7. Immunofluorescence

To determine the activity level of osteoclasts, immunofluorescence was utilized. The cells were isolated from human femoral heads and plated at 1 × 10^6^ cells/mL on 18 mm diameter rounded coverslips. On day 14, the media were aspirated, and the cells were washed with ice-cold 1X PBS and fixed with 4.4% PFA for 20 min at room temperature. The cells were washed with ice-cold 1X PBS and permeabilized for 10 min using 0.1% saponin (Sigma-Aldrich, St. Louis, MO, USA) diluted in 1X PBS on ice. After, non-specific binding was prevented by adding 3% bovine serum albumin (BSA, Fisher Scientific, Pittsburgh, PA, USA) diluted in 1X PBS and supplemented with 0.1% saponin for 1 h on ice. Cells from the control and M-CSF + RANKL groups were then treated with rabbit polyclonal anti-TRAP (Lot #C0314, Santa Cruz Biotechnology, Dallas, TX, USA) and goat polyclonal anti-Cathepsin K (Lot #J1613, Santa Cruz Biotechnology, Dallas, TX, USA) primary antibodies diluted at 1:100 in 1X PBS supplemented with 3% BSA and 0.1% saponin for 1 h on ice. The secondary control group was not incubated with primary antibodies. After 1 h, the cells were washed with 1X PBS on ice. All of the experimental groups were then treated with chicken-anti-rabbit (Alexa Fluor^TM^488, Catalog #A21441, Invitrogen, Eugene, OR, USA) and donkey-anti-goat (Alexa Fluor^TM^568, Catalog #A11057, Invitrogen, Eugene, OR, USA) secondary antibodies diluted at 1:500 in 1X PBS supplemented with 3% BSA and 0.1% saponin in the dark for 1 h on ice. The cells were washed for 5 min with 1X PBS on ice and the nuclei were stained using Hoechst 33342 (Catalog #AR0039, Bolster Bio, Pleasanton, CA, USA) for 8 min at room temperature away from the light. The coverslips were washed with 1X PBS on ice and the coverslips were mounted on glass slides with Cytoseal^TM^ (Thermo Fisher Scientific, Waltham, MA, USA) and allowed to dry for two days. The slides were then imaged using the Zeiss LSM880 with Airyscan Confocal Microscope (Wolf Hall, University of Delaware, Newark, DE, USA) using 20× and 63× objective lenses. At least 10 representative images were obtained from each group and processed using ImageJ. All data were normalized to the secondary control. Experiments were conducted with three OA patients and were repeated in triplicate.

### 2.8. Statistical Analysis

Data are displayed as mean + standard error of the mean (STE). Bar graphs were constructed to display the osteoclast percentage of total cells present in each group. “*” denotes statistical significance, where *p* is set to 0.05. All of the statistical analyses were conducted using Students’ *t*-test followed by the Tukey−Kramer HSD test. All outliers were removed using the Chauvenet’s Criterion method.

## 3. Results

### 3.1. RAW264.7 Cells Do Not Differentiate Readily into Osteoclasts

**Figure 3:**
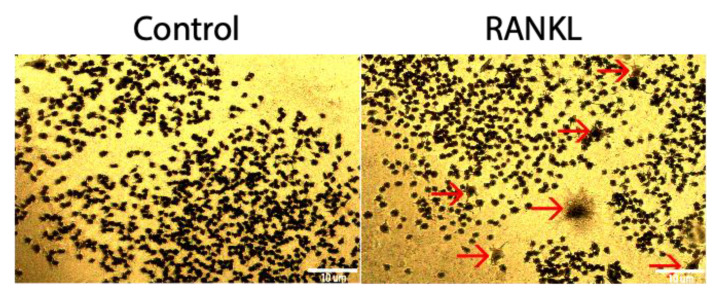
TRAP assay of RAW264.7 cells stimulated with RANKL. After 5 days of treatment with 10 ng/mL of RANKL, 2.5% of stimulated cells stained positive for TRAP, whereas 0.09% of control cells not treated with RANKL stained positive for TRAP. Red arrows designate a TRAP positive cell. Images were acquired with a 10× objective lens and scale bars are set to 10 μm. All of the experiments were conducted in triplicate.

RAW264.7 cells are murine monocytes/macrophages that can be differentiated into osteoclasts. However, the extent of osteoclastogenesis is not clear, and this cell line may be utilized to observe osteoclast activity [16]. To determine if RAW264.7cells could be differentiated into osteoclasts using 10 ng/mL of RANKL, a TRAP assay was utilized. Compared to the control group that was not stimulated with RANKL, the RANKL stimulated group was not significantly higher, as only 2.5% of cells stained positive for TRAP (Figure 3).

### 3.2. Optimal Conditions for Osteoclastogenesis Utilizing M-CSF and RANKL

**Figure 4:**
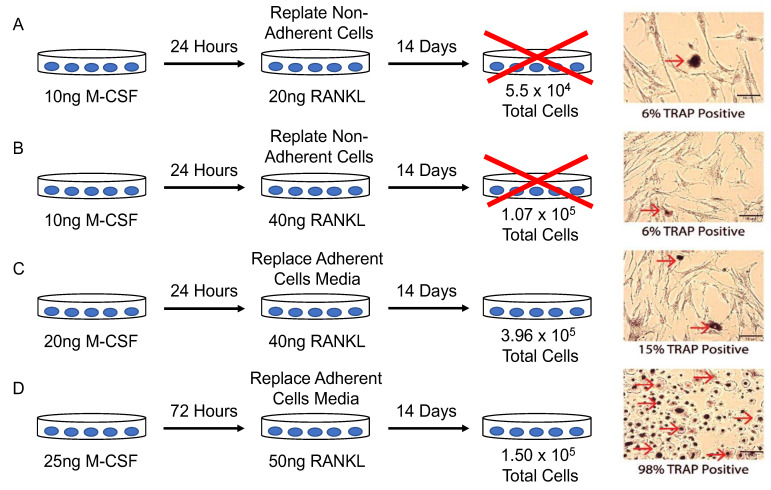
Cells extracted from human femoral heads were plated with different conditions and were stained for TRAP. (**A**) Cells were plated with 10 ng/mL M-CSF for 24 h. Non-adherent cells were replated at 5.5 × 10^4^ total cells per well and supplemented with 10 ng/mL M-CSF and 20 ng/mL RANKL. (**B**) Cells were plated with 10 ng/mL M-CSF for 24 h. Non-adherent cells were replated at 1.7 × 10^5^ total cells per well and supplemented with 10 ng/mL M-CSF and 40 ng/mL RANKL. (**C**) Cells were plated at 1.31 × 10^5^ total cells and supplemented with 20 ng/mL M-CSF for 24 h. Adherent cells were supplemented with fresh media with 20 ng/mL M-CSF and 40 ng/mL RANKL for 13 days. (**D**) Cells were plated at a density of 1.50 × 10^5^ cells/mL and supplemented with α-MEM with 25 ng/mL M-CSF for 3–4 days. After, the media of adherent cells was replaced with fresh α-MEM along with 25 ng/mL and 50 ng/mL RANKL. Cells were stained with tartrate resistant acid phosphatase (TRAP) and imaged to identify functional osteoclasts. Red arrows indicate TRAP-positive cells. Experiments were conducted in triplicate for at least five different patients. Images were acquired using the Nikon Eclipse TE300 epifluorescence microscope with a 10× objective. Scale bars are set at 10 μm.

Because RAW264.7 cells did not differentiate readily into osteoclasts in the present study, a different model was explored to observe osteoclast activity. While mouse models are available, a human model may be more reliable to study human osteoclastogenesis. Therefore, to obtain a different model for observing osteoclastogenesis, osteoclasts were extracted from the femoral heads of patients diagnosed with OA. After, the osteoclasts were plated at various concentrations and treated with differing amounts of M-CSF or RANKL to determine the optimum conditions for osteoclastogenesis. The optimized conditions included plating pre-osteoclasts at a density of 1.5 × 10^5^ cells/mL with 25 ng/mL M-CSF in α-MEM for 3 days. After 3 days, the cells were given fresh α-MEM with 25 ng/mL and 50 ng/mL RANKL for 11 days, and media were refreshed every 3–4 days. TRAP staining demonstrated that 98% of cells stained positive for TRAP (Figure 4D). However, in other conditions, the cells were stained 6% (Figure 4A), 6% (Figure 4B), and 15% (Figure 4C) positive for TRAP.

### 3.3. Cells isolated from Female and Male OA Patients Differentiate into Functional Osteoclasts

**Figure 5:**
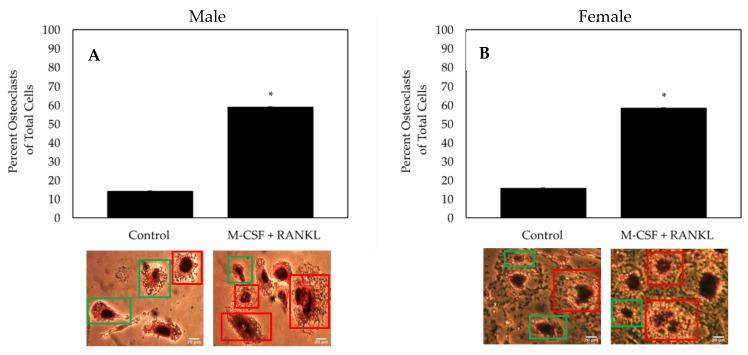
TRAP assay of the cells isolated from the femoral heads of OA patients. (**A**) Osteoclast count of male patients (*N* = 5). (**B**) Osteoclast count of female patients (*N* = 5). After stimulating cells for 14 days with M-CSF and RANKL, or M-CSF only, osteoclasts were identified as cells that stained positive for TRAP and comprised of three or more nuclei. Representative images are displayed underneath bars. Red boxes designate TRAP positive cells with more than three nuclei and green boxes denote macrophages/monocytes. Images were acquired with a 20× objective lens and all experiments were conducted in triplicate. “*” denotes statistical significance, where *p* is set to 0.05.

As both osteoclasts and macrophages can express TRAP, multinucleated cells must be identified to confirm the formation of an osteoclast. Here, TRAP and hematoxylin were utilized to measure the formation of osteoclasts. Furthermore, as OA affects both men and women, we conducted experiments with both genders. To determine the effectiveness of the differentiating cells isolated from female and male OA patients into osteoclasts, cells were treated with M-CSF and RANKL or M-CSF only (control). As indicated by TRAP positive and multinucleated cells, we demonstrated that ~60% of cells stimulated with RANKL differentiated into osteoclasts, whereas only ~15% of control cells differentiated into osteoclasts and most remained monocytes/macrophages (Figure 5). Furthermore, isolated cells from both male and female patients respond similarly to treatment.

### 3.4. Cells Isolated from Female and Male OA Patients Are Viable and Do Not Proliferate

**Figure 6:**
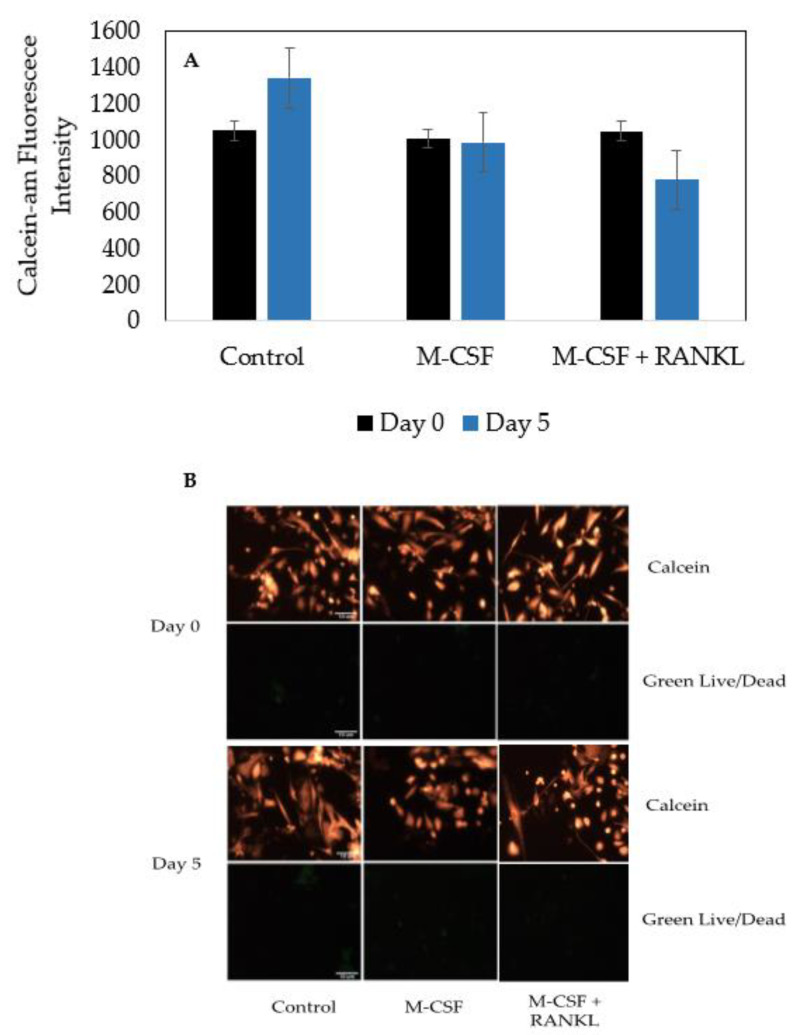
Calcein-AM-red-orange and Green Live/Dead staining for proliferation and viability. (**A**) Graphical representation of Calcein-AM fluorescence in different conditions. (**B**) Visual representations of Calcein-AM and Live/Dead stains. Cells were counted for 5 days, and immunofluorescence was captured using an epifluorescent microscope. Calcein-AM is indicative of the total number of viable cells, whereas green indicates an unviable cell. Scale bars are set to 10 μm. All experiments were conducted in triplicate and images were processed using ImageJ.

Osteoclasts are terminally differentiated cells derived from HSCs. Furthermore, data demonstrating the viability of bone marrow cells isolated from human femoral heads are unclear. Therefore, to assess the viability and terminal differentiation to osteoclasts, cells were incubated with Green Live/Dead stains and Calcein-AM-red-orange stains. After five days, the cells in the control, M-CSF only, and M-CSF + RANKL experimental groups did not exhibit proliferation (Figure 6A). Furthermore, as displayed by the very expression of the Green Live/Dead stain, the cells were viable and healthy in each group (Figure 6B).

### 3.5. Osteoclasts Isolated from OA Patients Express TRAP and Cathepsin K

**Figure 7:**
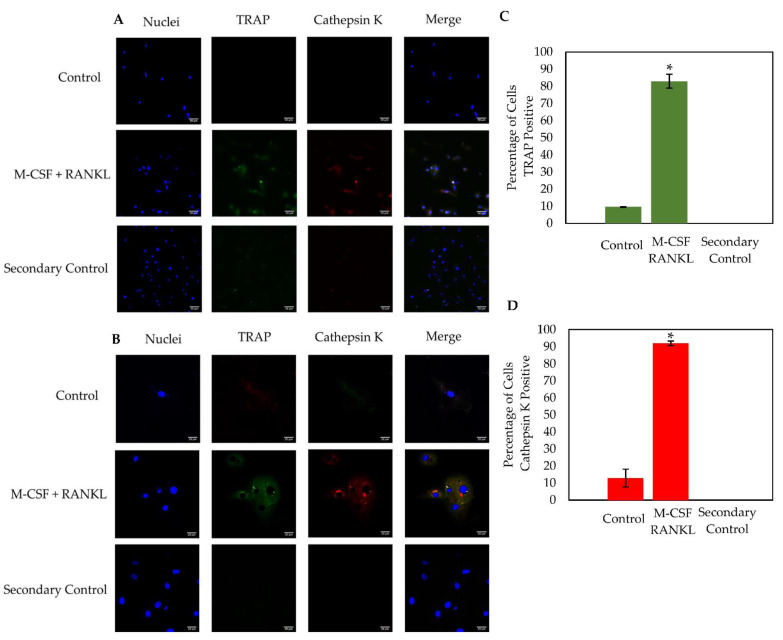
Immunostaining of cells isolated from male (*N* = 2) and female (*N* = 1) OA patients. Confocal microscopy was utilized to image cells at 20× (**A**) and 63× (**B**) magnification. The population of cells stimulated with M-CSF + RANKL was ~60%, whereas the control group had ~15% osteoclasts. As displayed in the figure, M-CSF + RANKL stimulated highly expressed TRAP and Cathepsin K cells, while the control cells produced very little of these osteoclast markers (**C**,**D**). Cells from 10 representative images obtained at random from all three patients were counted. The RANKL stimulated cells expressed significantly higher TRAP and Cathepsin K than the control groups (**C**,**D**). Experiments were completed in triplicate and processed using ImageJ. All fluorescence was normalized to the secondary control. “*” denotes statistical significance, where *p* is set to 0.05.

Osteoclasts express active enzymes that are responsible for degrading old or damaged bone. The most notable osteoclast markers are TRAP and Cathepsin K, as both as are highly expressed during resorption. Here, the osteoclast markers TRAP and Cathepsin K were immunostained in cells stimulated with RANKL or were left unstimulated. Cells were imaged using confocal microscopy at 20× and 63× magnification (Figure 7A,B). Similar to the TRAP stain, the RANKL experimental group contained ~60% osteoclasts while the control group displayed ~15% osteoclasts (data not shown). Furthermore, cells stimulated with RANKL expressed high levels of both TRAP and Cathepsin K, while this expression was minimal in the control cells (Figure 7C,D). At 63× magnification, high levels of TRAP and Cathepsin K were expressed in distinct domains within the RANKL-stimulated osteoclast, but this expression was seen very minimally in the control group, which is consistent with previous results (Figure 7B) [49,50,51].

## 4. Discussion

Proper formation of the skeleton during development is crucial for mobility and the protection of organs. The formation of new bone and the degradation of old or damaged bone is regulated by mononuclear osteoblasts and multinucleated osteoclasts [1,52,53]. Irregular activities of these bone cells can lead to juvenile osteoporosis in childhood, or osteopenia and osteoporosis later in adulthood [5,54]. While it is suggested that these bone disorders may arise due to hyperactivity of osteoclasts, the function of these bone resorption cells isolated directly from humans is not established [1,18]. Therefore, there is an urgent need to establish a reliable method of isolating viable bone marrow cells that can be differentiated into osteoclasts.

It has been demonstrated previously that RAW 264.7 cells can differentiate into osteoclasts [16]. However, the applications or extent of osteoclastogenesis of these cells is unclear. Therefore, we first demonstrate that RAW264.7 cells can be differentiated into osteoclasts, but very minimally (Figure 3). When stimulated with RANKL, only 2.5% of these cells expressed TRAP, indicating most cells remained monocytes/macrophages (Figure 3). These cells may continue proliferating into additional monocytes/macrophages, but do not readily terminally differentiate into osteoclasts at day 5, which could explain the minimal TRAP expression [55].

To differentiate cells into osteoclasts to study bone disorders, we utilized femoral heads from patients diagnosed with OA after undergoing total hip replacement surgery. Human femoral heads are readily available due to the increasing occurrence of hip replacement surgery. These femoral heads provide an opportunity to study osteoclastogenesis with cells isolated directly from the bone microenvironment. Isolating cells from this microenvironment provides a useful tool to study the functions of osteoclasts within their natural bone environment. We observed that the removal of the trabecular bone from the femoral heads and washing interior surface provided cells that could be differentiated into osteoclasts [56,57]. While this method has been used in previous research, it is unclear whether these cells isolated directly from the human bone can be differentiated into functional osteoclasts after being stimulated with M-CSF and RANKL [40,58,59]. Previous studies demonstrate that PBMCs can be obtained in large quantities to study osteoclastogenesis; however, utilizing cells directly from the bone marrow may be a useful alternative approach. Here, we provide optimal conditions for osteoclastogenesis that produced functional osteoclasts (Figure 4). This method provides an isolation technique directly from femoral heads that can produce enzymatically active osteoclasts, especially in other bone disorders such as osteoporosis.

While 98% of the isolated cells stained positive for TRAP, it has been illustrated in previous research that monocytes/macrophages and osteoclasts can express this protein [51,60]. For nuclei identification, we utilized hematoxylin, which binds to DNA and fluoresces blue [61,62]. In the current study, the population of cells stimulated with RANKL was ~60% osteoclasts (Figure 5). This group displayed a significantly higher percentage of osteoclasts when compared to the control group (~15% osteoclasts; Figure 5). Notably, although not shown, there was no significant difference between the osteoclast percentage between both genders (*N* = 10). Thus, it is suggested that the mechanisms of osteoclastogenesis within OA patients are not affected by gender. Moreover, these data suggest that cells isolated from human femoral heads can be differentiated into functional osteoclasts and this method may be utilized to study their activity in OP.

Finally, to assess the functionality of differentiated osteoclasts, cells were immunostained for the osteoclastic markers TRAP and Cathepsin K [63]. Both enzymes are highly expressed during bone resorption and are key players in bone turnover [64]. We utilized confocal microscopy to obtain images of the cells stimulated with RANKL or left unstimulated. Here, we showed that distinct osteoclasts formed and expressed high levels of TRAP and Cathepsin K compared to the control groups (Figure 7). Furthermore, it has been demonstrated that monocytes/macrophages express low levels of TRAP and Cathepsin K, which is consistent with the current study (Figure 7A) [50,51,59,60]. These results indicate that cells differentiated directly from human femoral heads can be treated with M-CSF and RANKL to become functional osteoclasts.

In conclusion, the objective of the current study was to obtain a reliable model of osteoclastogenesis to study bone disorders. Our data demonstrated that cells isolated from human femoral heads are superior to study osteoclastogenesis when compared to RAW264.7 cells (Figure 3 and Figure 4). Furthermore, we demonstrated that cells isolated from human femoral heads became multinucleated cells and stained positive for TRAP (Figure 5). Isolated cells were viable and did not proliferate, indicating that the RANKL stimulated cells were terminally differentiated into osteoclasts (Figure 6). Finally, RANKL stimulated cells stained positive for both TRAP and Cathepsin K, along with containing more than two nuclei, indicating that they were functional osteoclasts (Figure 7). Taken together, our data provide a reliable method for obtaining functional osteoclasts from human femoral heads. While this current study has provided an optimized method for generating osteoclasts from human femoral heads, future studies should delineate the resorptive activity of these cells to confirm the Cathepsin K and TRAP expression. These data can assist future work in elucidating the precise role of osteoclasts in bone disorders, such as osteopenia and osteoporosis.

## Data Availability

All data are contained within the article.
